# Identifying cooperative transcription factors in yeast using multiple data sources

**DOI:** 10.1186/1752-0509-8-S5-S2

**Published:** 2014-12-12

**Authors:** Fu-Jou Lai, Mei-Huei Jhu, Chia-Chun Chiu, Yueh-Min Huang, Wei-Sheng Wu

**Affiliations:** 1 Department of Engineering Science, National Cheng Kung University, No.1 University Road, 70101, Tainan, Taiwan; 2 Department of Electrical Engineering, National Cheng Kung University, No.1 University Road, 70101, Tainan, Taiwan

**Keywords:** transcription factor cooperativity, nucleosome, transcription factor binding site, yeast

## Abstract

**Background:**

Transcriptional regulation of gene expression is usually accomplished by multiple interactive transcription factors (TFs). Therefore, it is crucial to understand the precise cooperative interactions among TFs. Various kinds of experimental data including ChIP-chip, TF binding site (TFBS), gene expression, TF knockout and protein-protein interaction data have been used to identify cooperative TF pairs in existing methods. The nucleosome occupancy data is not yet used for this research topic despite that several researches have revealed the association between nucleosomes and TFBSs.

**Results:**

In this study, we developed a novel method to infer the cooperativity between two TFs by integrating the TF-gene documented regulation, TFBS and nucleosome occupancy data. TF-gene documented regulation and TFBS data were used to determine the target genes of a TF, and the genome-wide nucleosome occupancy data was used to assess the nucleosome occupancy on TFBSs. Our method identifies cooperative TF pairs based on two biologically plausible assumptions. If two TFs cooperate, then (i) they should have a significantly higher number of common target genes than random expectation and (ii) their binding sites (in the promoters of their common target genes) should tend to be co-depleted of nucleosomes in order to make these binding sites simultaneously accessible to TF binding. Each TF pair is given a cooperativity score by our method. The higher the score is, the more likely a TF pair has cooperativity. Finally, a list of 27 cooperative TF pairs has been predicted by our method. Among these 27 TF pairs, 19 pairs are also predicted by existing methods. The other 8 pairs are novel cooperative TF pairs predicted by our method. The biological relevance of these 8 novel cooperative TF pairs is justified by the existence of protein-protein interactions and co-annotation in the same MIPS functional categories. Moreover, we adopted three performance indices to compare our predictions with 11 existing methods' predictions. We show that our method performs better than these 11 existing methods in identifying cooperative TF pairs in yeast. Finally, the cooperative TF network constructed from the 27 predicted cooperative TF pairs shows that our method has the power to find cooperative TF pairs of different biological processes.

**Conclusion:**

Our method is effective in identifying cooperative TF pairs in yeast. Many of our predictions are validated by the literature, and our method outperforms 11 existing methods. We believe that our study will help biologists to understand the mechanisms of transcriptional regulation in eukaryotic cells.

## Background

Transcriptional regulation plays a crucial role in the regulation of gene expression. As well known, it is accomplished by the binding of transcription factors (TFs) to the TF binding sites (TFBSs) in the promoters of genes. In eukaryotic cells, transcriptional regulation is usually achieved by the cooperation between multiple TFs to regulate the expression of genes. Therefore, knowing the precise cooperative interactions among TFs is helpful for uncovering the mechanisms of transcriptional regulation.

With advances in high-throughput microarray technologies and diverse data sources, it is now possible to investigate the cooperative interactions among TFs. Many computational methods have been developed to identify cooperative TF pairs by using one or several kinds of experimental data. Some methods only used ChIP-chip data [1-3]. Several other methods integrated ChIP-chip and gene expression data [4-7]. Another two methods integrated ChIP-chip data with other data sources such as protein-protein interaction data [8] and TF knockout data [9]. On the contrary, Pilpel et al.'s method did not use ChIP-chip data but integrated TFBS and gene expression data [10]. Wang et al.'s and Hu et al.'s methods both integrated multiple data sources by using a Bayesian approach [11,12].

ChIP-chip, gene expression, TFBS, TF knockout and protein-protein interaction data were used to investigate the cooperative interactions among TFs in the above mentioned methods. However, the nucleosome occupancy data was not used even though several researches have revealed the association between nucleosomes and TFBSs [13-16]. Because nucleosome occupancy has been demonstrated as an important strategy to regulate gene expression by affecting the accessibility of TFBSs to TFs, this biological knowledge motivates us to consider the effect of nucleosome occupancy on the cooperativity between TFs and adopt the nucleosome occupancy data for our research. Our method is developed based on the following two rationales. First, if two TFs cooperate, they should have a significantly higher number of common target genes than random expectation. Second, the TFBSs of these two cooperative TFs (in the promoters of their common target genes) should tend to be co-depleted of nucleosomes in order to make themselves simultaneously accessible to TF binding.

## Methods

### Data sources

We used three data sources in this study. First, the genome-wide nucleosome occupancy data of Saccharomyces cerevisiae was downloaded from Mavrich et al.'s study [17]. They established a genome-wide map of nucleosome locations and the map shows which region in the genome is occupied by nucleosomes. Second, the TF-gene documented regulation data was downloaded from YEASTRACT database [18], which deposited the documented regulation evidence (from the ChIP-chip and TF knockout experiments in the literature) between TFs and their target genes. Third, the TFBS data was downloaded from SwissRegulon database [19]. Each TFBS has its predicted genomic location and a posterior probability to indicate the confidence of this putative TFBS. In this study, a threshold 0.3 of the posterior probability was applied to select putative TFBSs. The total number of distinct TFs from the above two databases was 186, and therefore 17205 (186*185/2) TF pairs were considered in this study.

### The proposed method

**Figure 1:**
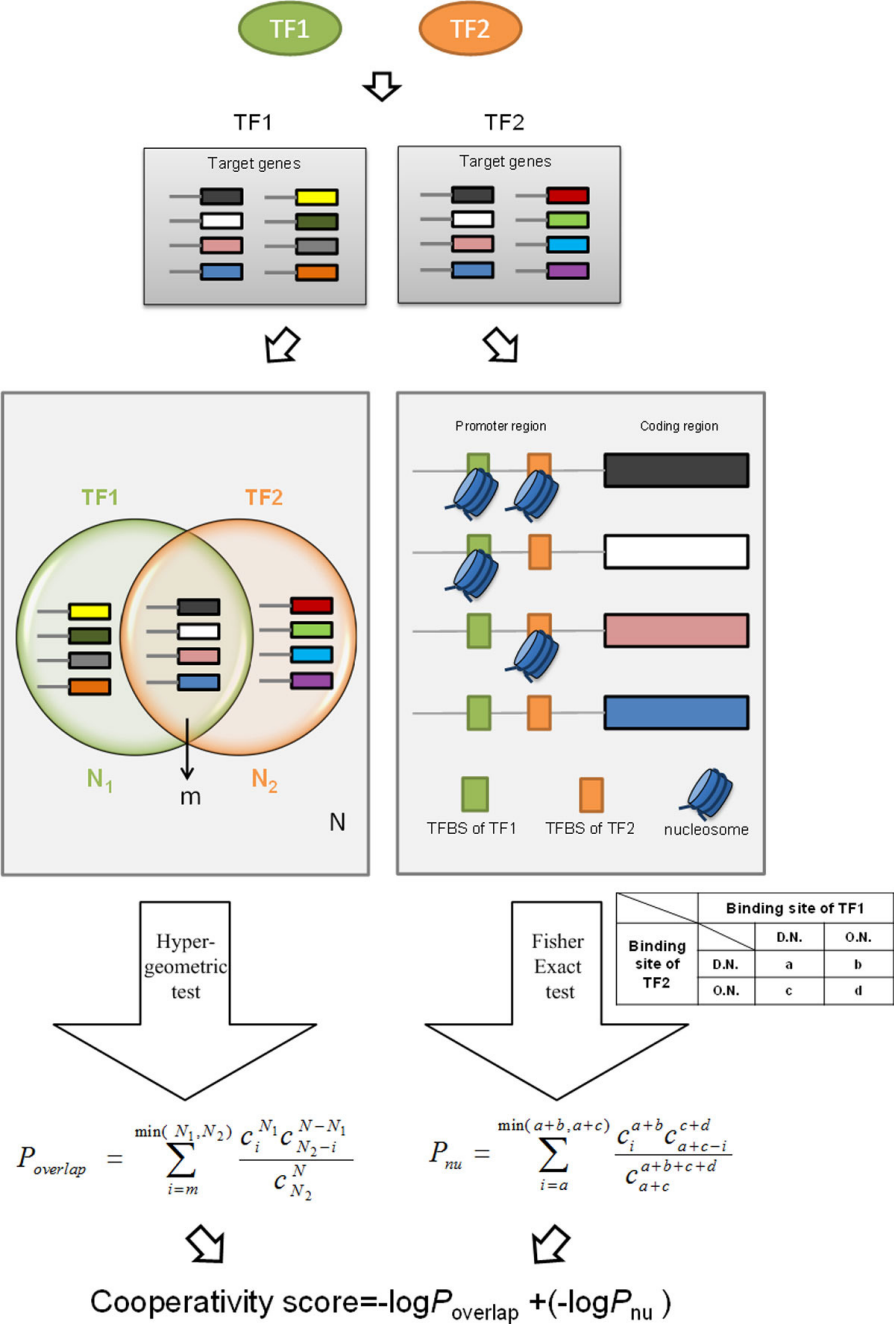
**Flowchart of our method**. The figure shows a schematic description of the steps used for determining the cooperativity score of two TFs. In the 2 × 2 contingency table, the D.N. stands for depletion of nucleosomes while O.N. stands for occupancy of nucleosomes.

The proposed method is developed based on the following two biologically plausible assumptions. First, two cooperative TFs should share a significantly larger set of target genes than random expectation. This assumption has also been used in existing methods [1,11,12,20]. Second, the TFBSs of two cooperative TFs (in the promoters of their common target genes) should be co-depleted of nucleosomes to make themselves simultaneously accessible to TF binding. This assumption is biologically plausible since it has been shown that functionally cooperative TF pairs are associated with nucleosome-depleted promoters [21]. Therefore, given a TF pair, we calculate the significance of the overlap of their target genes and the significance of being co-depleted of nucleosomes on their TFBSs. Our method assigns a cooperativity score to each of the 17205 TF pairs. Finally, 27 TF pairs whose cooperativity scores larger than 120 are predicted as cooperative TF pairs. The flow chart of our method is shown in Figure 1 and described as follows.

#### Step1-Define the target genes of each of the 186 TFs

A TF's target genes are defined as those genes (i) that are known to be regulated by the TF from the TF-gene documented regulation evidence (retrieved from YEASTRACT database [18]) or (ii) whose promoters contain the binding sites of the TF (retrieved from SwissRegulon database [19]).

#### Step2-Calculate the significance of the target gene overlap

Given a TF pair, the significance of the overlap between the target genes of these two TFs is calculated using the Hyper-geometric test [22]:

(1) $$P_{overlap}=\sum_{i=m}^{\min(N_{1},N_{2})}\frac{\left(\begin{array}{c} N_{1}\\ i \end{array}\right)\left(\begin{array}{c} N-N_{1}\\ N_{2}-i \end{array}\right)}{\left(\begin{array}{c} N\\ N_{2} \end{array}\right)}$$

where *N*_1 _is the number of target genes of the first TF, *N*_2 _is the number of target genes of the second TF, *N *= 6576 is the number of total genes in the yeast genome, and *m *is the number of common target genes. The smaller the *P_overlap_*, the more significant the target gene overlap.

#### Step3-Calculate the significance of being co-depleted of nucleosomes on the TFBSs

From the common target genes of the given TF pair, we extract the genes which have both TFs' binding sites in their promoters, and denote it as set *A*. If multiple TFBSs of the same TF are found, only the most confident TFBS (with the highest posterior probability) is considered. Therefore, the promoter of each gene in set *A *contains one TFBS of the first TF and one TFBS of the second TF.

For each gene in set *A*, the state of nucleosome occupancy of each of the two TFBSs can be specified to one of the two categories:

$$\left\{\begin{array}{c} Occupied,\text{if}\;\text{any}\;\text{position}\;\text{in}\;\text{the}\;\text{TFBS}\;\text{is}\;\text{occupied}\;\text{by}\;\text{anucleosome}.\\ Depleted,\text{if}\;\text{no}\;\text{position}\;\text{in}\;\text{the TFBS}\;\text{is}\;\text{occupied}\;\text{by}\;\text{anucleosome}. \end{array}\right)$$

Then the state of nucleosome occupancy can be converted to a binary value and denoted as *S*_(*TFBSj*)_:

$$S_{\left(TFBSj\right)}=\left\{\begin{array}{cc} 1, & \text{occupied}\\ 0, & \text{depleted} \end{array}\right)$$

where *j *= 1 for the first TF and *j *= 2 for the second TF. According to the values of *S*_(*TFBS*1) _and *S*_(*TFBS*2)_, each gene in set *A *can be assigned to a cell in a 2 × 2 contingency table (see Figure 1) and the number of genes in each of the four cells in the contingency table can be obtained. Then the Fisher exact test [23] is used to calculate the significance of *TFBS*1 and *TFBS*2 to be co-depleted of nucleosomes. The *p*-value is denoted as

(2) $$P_{nu}=\overset{min\left(a+b,a+c\right)}{\underset{i=a}{\sum }}\frac{\left(\begin{array}{c} a+b\\ i \end{array}\right)\left(\begin{array}{c} c+d\\ a+c-i \end{array}\right)}{\left(\begin{array}{c} a+b+c+d\\ a+c \end{array}\right)}$$

where *a *is the number of genes whose promoters contain both *TFBS*1 and *TFBS*2 depleted of nuclesomes, *b *is the number of genes whose promoters contain *TFBS*1 occupied by nucleosomes and *TFBS*2 depleted of nuclesomes, *c *is the number of genes whose promoters contain *TFBS*1 depleted of nucleosomes and *TFBS*2 occupied by nuclesomes, and *d *is the number of genes whose promoters contain both *TFBS*1 and *TFBS*2 occupied by nuclesomes. The smaller the *P_nu_*, the more significant the *TFBS*1 and *TFBS*2 to be co-depleted of nucleosomes.

#### Step4-Calculate the cooperativity score

The cooperativity score is defined as *−logP_overlap _*+(*−logP_nu_*). The higher the score is, the more likely a TF pair has cooperativity. There are two situations that can have a high score. One is a small *P_overlap_*, i.e. the TF pair has a significant overlap between their target genes. The other situation is a small *P_nu_*, i.e. the two TFs show a high tendency of being co-depleted of nucleosomes on their TFBSs to make them simultaneously accessible to TF binding.

## Results

According to the cooperativity score, 17205 TF pairs can be sorted decreasingly, and then constitute a ranked prediction of cooperative TF pairs. Finally, 27 TF pairs whose cooperativity scores larger than 120 are predicted as cooperative TF pairs.

### Detailed investigation of the 27 predicted cooperative TF pairs

In Table 1, we list these 27 predicted cooperative TF pairs. All of them have at least one of the following three lines of evidence: (i) being predicted by existing methods, (ii) the existence of protein-protein interactions, and (iii) the co-annotation in the same MIPS functional categories. More precisely, 63% (17/27) of the pairs have all three lines of evidence, 26% (7/27) of the pairs have two lines of evidence, and 11% (3/27) of the pairs have only one line of evidence.

**Table 1:** The 27 predicted cooperative TF pairs

Ranking	TF1	TF2	Consistent with existing methods' predictions	Having PPIs?	Co-annotated MIPS functional categories
1	Reb1	YDR026C	[3]	--	--
2	Fkh2	Fkh1	[1],[2],[3],[4],[7],[8],[38]	Y	mitotic cell cycle and cell cycle control; budding, cell polarity and filament formation
3	Tye7	Cbf1	[3]	Y	metabolism
4	Dig1	Ste12	[3],[7],[20]	Y	protein folding; pheromone response, mating-type determination, sex-specific proteins; budding, cell polarity and filament formation
5	Pdr3	Pdr1	---	Y	DNA binding; chemical agent resistance; detoxification
6	Swi6	Mbp1	[2],[3],[4],[5],[7],[20],[38]	Y	DNA synthesis and replication; mitotic cell cycle and cell cycle control; protein with binding function or co-factor requirement (structural or catalytic)
7	Swi6	Swi4	[2],[3],[4],[5],[7],[9],[20],[38]	Y	G1/S transition of mitotic cell cycle
8	Dal80	Gln3	---	Y	regulation of nitrogen metabolism
9	Gln3	Gat1	[3]	Y	regulation of nitrogen metabolism
10	Dal80	Gat1	---	--	regulation of nitrogen metabolism
11	Pho4	Cbf1	---	Y	metabolism; DNA binding
12	Pho4	Tye7	---	--	metabolism
13	Hap3	Hap2	[20]	Y	regulation of C-compound and carbohydrate metabolism
14	Gal80	Gal4	---	Y	regulation of C-compound and carbohydrate metabolism
15	Met31	Met32	[3]	Y	metabolism of methionine; metabolism of cysteine; regulation of amino acid metabolism; regulation of nitrogen, sulfur and selenium metabolism; DNA binding
16	Msn4	Msn2	[3]	Y	DNA binding; stress response
17	Rap1	Fhl1	[3],[8],[20]	Y	---
18	Tec1	Ste12	[3]	Y	budding, cell polarity and filament formation
19	Swi6	Stb1	[3],[7]	Y	G1/S transition of mitotic cell cycle
20	Hap2	Hap5	[8]	Y	regulation of C-compound and carbohydrate metabolism
21	Nrg1	Nrg2	---	Y	protein with binding function or co-factor requirement (structural or catalytic); budding, cell polarity and filament formation
22	Hap3	Hap5	[8],[20]	Y	regulation of C-compound and carbohydrate metabolism
23	Swi4	Stb1	[3],[4],[7]	Y	G1/S transition of mitotic cell cycle
24	Swi5	Ace2	[2],[3],[4],[7],[9]	Y	G1 phase of mitotic cell cycle
25	Sok2	Phd1	[3]	Y	budding, cell polarity and filament formation
26	Hap5	Hap4	---	Y	regulation of C-compound and carbohydrate metabolism
27	Mbp1	Swi4	[1],[2],[3],[6],[7],[9]	Y	mitotic cell cycle and cell cycle control

Note that among these 27 predicted cooperative TF pairs, 19 pairs are also predicted by existing methods. The other 8 pairs are novel cooperative TF pairs predicted by our method. The biological relevance of these 8 novel cooperative TF pairs is justified by the existence of protein-protein interactions and co-annotation in the same MIPS functional categories. More precisely, 75% (6/8) of the novel pairs

(i.e. Pdr1-Pdr3, Dal80-Gln3, Pho4-Cbf1, Gal80-Gal4, Nrg1-Nrg2 and Hap5-Hap4) are highly biologically plausible since they have protein-protein interactions and are co-annotated in the same MIPS functional categories, and the other 25% (2/8) of the novel pairs (i.e. Dal80-Gat1 and Pho4-Tye7) are moderately biologically plausible since they are co-annotated in the same MIPS functional categories but do not have protein-protein interactions.

### Performance comparison with 11 existing methods

In this study, we adopted three performance indices to compare our predictions with 11 existing methods' predictions (Table 2). Depending on the threshold value used, different methods obtained different number of predicted cooperative TF pairs (PCTFPs). The three performance indices are introduced in following subsections, and the comparison results are also shown.

**Table 2:** The 11 compared existing methods.

Existing methods	Data sources used	Method description	Threshold of p-value	Number of predicted cooperative TF pairs
Wang et al. (2006) [38]	ChIP-chip data, gene expression data, TFBS data	They developed a new framework to infer the combinatorial control of TFs by integrating heterogeneous functional genomic datasets.	10 ^−3^	14

Tsai et al. (2005) [5]	ChIP-chip data, gene expression data	They used statistical methods to identify yeast cell cycle TFs and synergistic pairs of TFs.	10 ^−5^	18

Elati et al. (2007) [28]	Gene expression data	They adopted a data mining system to learn transcriptional regulation relationship from gene expression data.	10 ^−3 ^	20

Nagamine et al. (2005) [8]	ChIP-chipdata, PPI data	They inferred the cooperative pairs under the assumption that the existence of interaction between two proteins suggests that they contribute to the same or similar biological process.	10 ^−3 ^	24

Datta and Zhao (2007) [2]	ChIP-chip data	They used a log-linear model to study cooperative binding among TFs and developed an Expectation-Maximization algorithm for statistical inferences.	10 ^−3 ^	25

He et al. (2006) [6]	ChIP-chip data, gene expression data	They adopted the microarray expression data to predict the cooperative TF pairs by testing whether the expression of target genes was significantly influenced by their cooperative effect with the multivariate method, ANOVA.	10 ^−2 ^	30

Banerjee and Zhang (2003) [4]	ChIP-chip data, gene expression data	They infer the cooperative pairs under the assumption that a pair of TFs is cooperative if genes regulated by both TFs are more co-expressed than those genes regulated by either TF alone.	5 × 10 ^−2 ^	31

Chang et al. (2006) [7]	ChIP-chip data, gene expression data	They employed a stochastic system model to assess TF cooperativity.	10 ^−21 ^	55

Yang et al. (2010) [9]	ChIP-chip data, TF knockout data	They predicted cooperativity between TFs by identifying the most statistically significant overlap of target genes regulated by two TFs in ChIP-chip data and TF knockout data.	5 × 10 ^−3^	186

Chen et al. (2012) [3]	ChIP-chip data	They facilitated identification of interactions between TFs by using motif discovery method when detecting overlapping targets of TFs based on ChIP-chip data.	10 ^−3^	221

Yu et al. (2006) [1]	ChIP-chip data	They proposed a method: Motif-PIE, which predicts interacting TF pairs by using a motif discovery procedure.	10 ^−8^	300

The table shows the information of 11 existing methods adopted for comparison. The data sources used, a brief description of the method, the p-value threshold and the number of predicted cooperative TF pairs of each method are described in columns 2, 3, 4 and 5.

#### Performance index 1: The similarity of protein-protein interaction partners between the two TFs of each PCTFP

**Figure 2:**
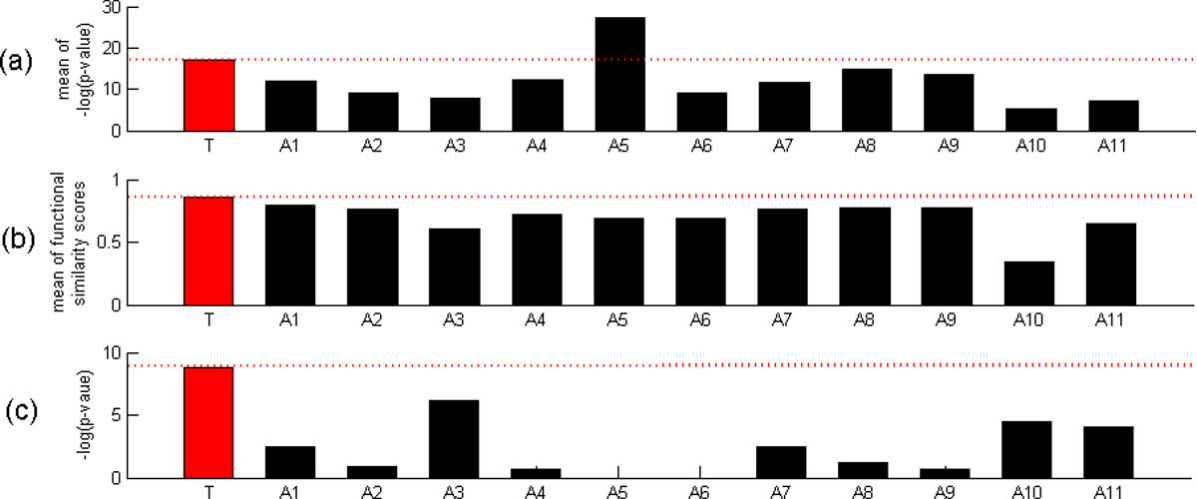
**Comparison of our method with 11 existing methods based on three performance indices**. This figures shows the comparison results of our methods with 11 existing methods using (a) the performance index 1, (b) the performance index 2, and (c) the performance index 3. Note that T stands for our method, A1 for Banerjee and Zhang' s method, A2 for Chang et al.'s method, A3 for Chen et al.'s method, A4 for Datta and Zhao's method, A5 for Elati et al.'s method, A6 for He et al.'s method, A7 for Nagamne et al.'s method, A8 for Tsai et al.'s method, A9 for Wang's method, A10 for Yang et al.'s method, and A11 for Yu et al.'s method.

Following previous studies in the literature [3,4,8,24], we evaluated cooperativity between two TFs in a PCTFP based on the rationale: the similarity of protein-protein interactions (PPI) partners between two TFs suggests that they contribute to the same biological processes and participate in the same regulatory mechanism. The physical PPI data were downloaded from the BioGRID database [25]. Given a list of PCTFPs from a method, we measured the similarity of PPI partners between the two TFs of each PCTFP by calculating a score *−logP *, which represents the significance of their PPI partners overlap. Note that *P *is the p-value calculated by the hypergeometric distribution shown in the formula (1), *N*_1 _is the number of genes whose proteins have physical PPI with the first TF, *N*_2 _is the number the genes whose proteins have physical PPI with the second TF, *N *= 6575 is the number of total genes in the yeast genome, and *m *is the number of genes whose proteins have physical PPI with both TFs. The greater the *−logP *is, the more significant the cooperativity of a PCTFP is. To evaluate the performance of a list of PCTFPs from a method, where each PCTFP has been given a score *−logP *, we took the mean of these scores as the final score of this performance index. Figure 2a shows that our method outperforms 10 existing methods in this score.

#### Performance index 2: Functional similarity between the two TFs of each PCTFP

We evaluated cooperativity between two TFs in a PCTFP based on the rationale: if two TFs have similar biological functions, then they tend to participate in the same regulatory mechanism [24]. The functional similarity score of a PCTFP is adopted from Yang et al.'s study [26], which proposed an improving GO semantic similarity measures using download random walks. The greater the functional similarity score is, the more significant the cooperativity of a PCTFP is. To evaluate the performance of a list of PCTFPs from a method, where each PCTFP has been given a functional similarity score, we took the mean of these scores as the final score of this performance index. Figure 2b shows that our method outperforms 11 existing methods in this score.

#### Performance index 3: The significance of the overlap between a list of PCTFPs from a method and a benchmark set of 27 known cooperative TF pairs

Yang et al. [9] proposed a performance index to test the prediction accuracy of different methods by comparing the significance of the overlap of different lists of PCTFPs with a benchmark set of known cooperative TF pairs. The benchmark set (Table 3) has 27 TF pairs, which is complied from the MIPS transcription complex catalog [27]. As far as we know, this is the only high-quality dataset of TF cooperativity currently available [9]. Then given a list of predicted cooperative TF pairs from a method, we calculated the significance of the overlap of this list with the benchmark set using Yang et al.'s index. For the given list, a score which represents the significance of the overlap is defined as the *−logP *, where *P *is the *p*-value computed using Fisher exact test. The higher the score, the better the performance. Figure 2c shows that our method outperforms 11 existing methods in this score.

**Table 3:** The benchmark set of 27 known cooperative TF pairs.

TF pairs	MIPS complex ID	MIPS complex name
ARG80-MCM1	510.190.120	ARG complex

MET4-MET28	510.190.160.10 510.190.160.20 510.190.160.30	Cbf1/Met4/Met28 complex Met4/Met28/Met31 complex Met4/Met28/Met32 complex

STP4-STP1	440.30.30	tRNA splicing

IME1-UME6	510.190.200	Ume6/Ime1 complex

HAP5-HAP4	510.160	CCAAT-binding factor complex

STP2-STP1	440.30.30	tRNA splicing

HAP2-HAP3	510.160	CCAAT-binding factor complex

ARG80-ARG81	510.190.120	ARG complex

MET4-MET31	510.190.160.20	Met4/Met28/Met31 complex

CBF1-MET28	510.190.160.10	Cbf1/Met4/Met28 complex

MCM1-ARG81	510.190.120	ARG complex

HAP5-HAP2	510.160	CCAAT-binding factor complex

HAP4-HAP2	510.160	CCAAT-binding factor complex

PIP2-OAF1	510.190.100	OAF complex

MET4-CBF1	510.190.160.10	Cbf1/Met4/Met28 complex

GCR1-GCR2	510.190.90	GCR complex

RTG1-RTG3	510.190.130	RTG complex

SWI6-MBP1	510.190.70	MBF complex

HAP5-HAP3	510.160	CCAAT-binding factor complex

HAP4-HAP3	510.160	CCAAT-binding factor complex

GAL3-GAL80	510.190.80	GAL80 complex

MET4-MET32	510.190.160.30	Met4/Met28/Met32 complex

STP4-STP2	440.30.30	tRNA splicing

SWI4-SWI6	510.190.60	SBF complex

GAL80-GAL4	510.190.80	GAL80 complex

MET32-MET28	510.190.160.30	Met4/Met28/Met32 complex

MET28-MET31	510.190.160.20	Met4/Met28/Met31 complex

The list of 27 known cooperative TF pairs are derived from biochemically well-defined transcriptional complexes within the MIPS complex catalogue.

## Discussion

### Our method is robust against different thresholds of the cooperativity score

**Figure 3:**
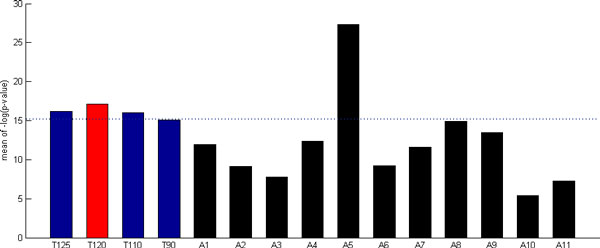
**The performance of our method when using different thresholds of the cooperativity score**. To check the robustness of our method against different thresholds of cooperativity scores, we tested four different thresholds (125, 120, 110 and 90). The figure shows that no matter which threshold is used, the performance of our method is always the same (i.e. superior to 10 out of 11 existing methods) on the performance index 1. This suggests that our method is robust against different thresholds of the cooperativity score.

In this study, we set a threshold of cooperativity score to be 120 and reported 27 PCTFPs whose cooperativity scores are larger than the threshold. The number of PCTFPs reported by our method is similar to those of five previous methods [2,4,6,8,28]. To check the robustness of our method against different thresholds of the cooperativity score, we tested four different thresholds (125, 120, 110 and 90). Figure 3 shows that no matter which threshold is used, the performance of our method is always the same (i.e. superior to 10 out of 11 existing methods) on the performance index 1. This suggests that our method is robust against different thresholds of the cooperativity score.

### Our method is robust against different qualities of TFBS data

**Figure 4:**
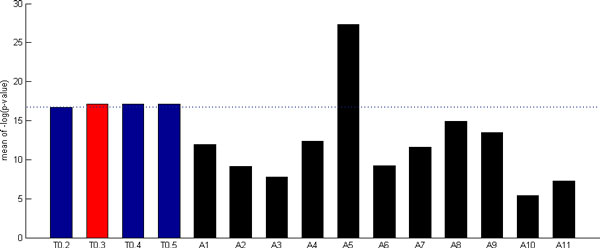
**The performance of our method when using the TFBS data with different qualities**. To check the robustness of our method against different TFBS qualities, we tested four different posterior probability thresholds (0.2, 0.3, 0.4 and 0.5). The figure shows that no matter which threshold is used, the performance of our method is always the same (i.e. superior to 10 out of 11 existing methods) on the performance index 1. This suggests that our method is robust against different thresholds of posterior probability to control the TFBS quality.

The quality of TFBS data retrieved from SwissRegulon database depends on the posterior probability threshold being used. In this study, a threshold 0.3 of the posterior probability was applied to select putative TFBSs. To check the robustness of our method against different qualities of TFBS data, we tested four different posterior probability thresholds (0.2, 0.3, 0.4 and 0.5). Figure 4 shows that no matter which threshold is used, the performance of our method is always the same (i.e. superior to 10 out of 11 existing methods) on the performance index 1. This suggests that our method is robust against different thresholds of posterior probability to control the quality of TFBS data.

### Our method outperforms existing methods in the precision and recall when using a benchmark set of 27 known cooperative TF pairs

**Figure 5:**
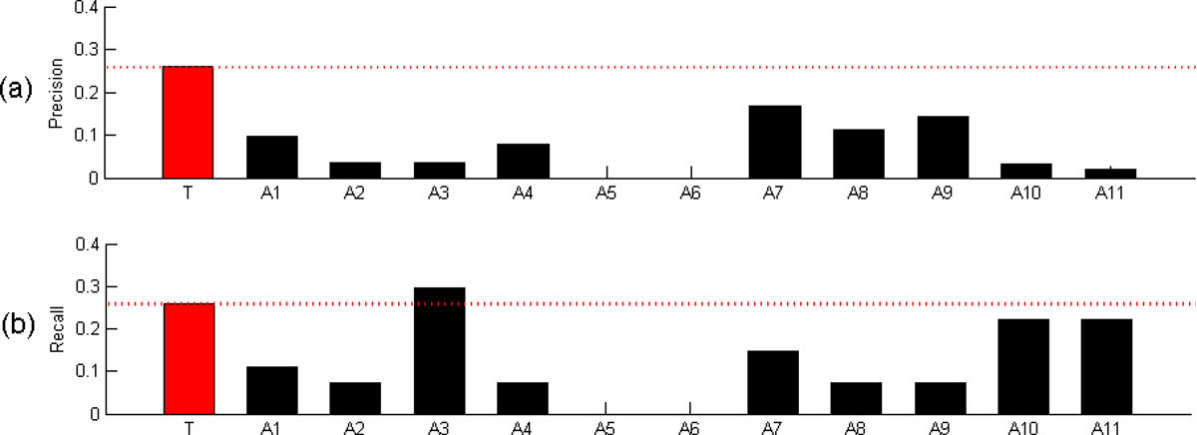
**Comparison of our method with 11 existing methods based on the precision and recall when using a benchmark set of 27 known cooperative TF pairs**. Here, we use the precision and recall to evaluate the performance of a method. The figure shows that our method outperforms 11 existing methods in the precision and outperforms 10 existing methdos in the recall.

In the performance index 3, the significance of the overlap of the list of PCTFPs from a method with the benchmark set of 27 known cooperative TF pairs is used to evaluate the performance of a method. Here, we use the precision and recall to evaluate the performance of a method. As shown in Figure 5, our method outperforms 11 existing methods in the precision and outperforms 10 existing methods in the recall.

### The nucleosome occupancy data contributes to the overall improved prediction

**Figure 6:**
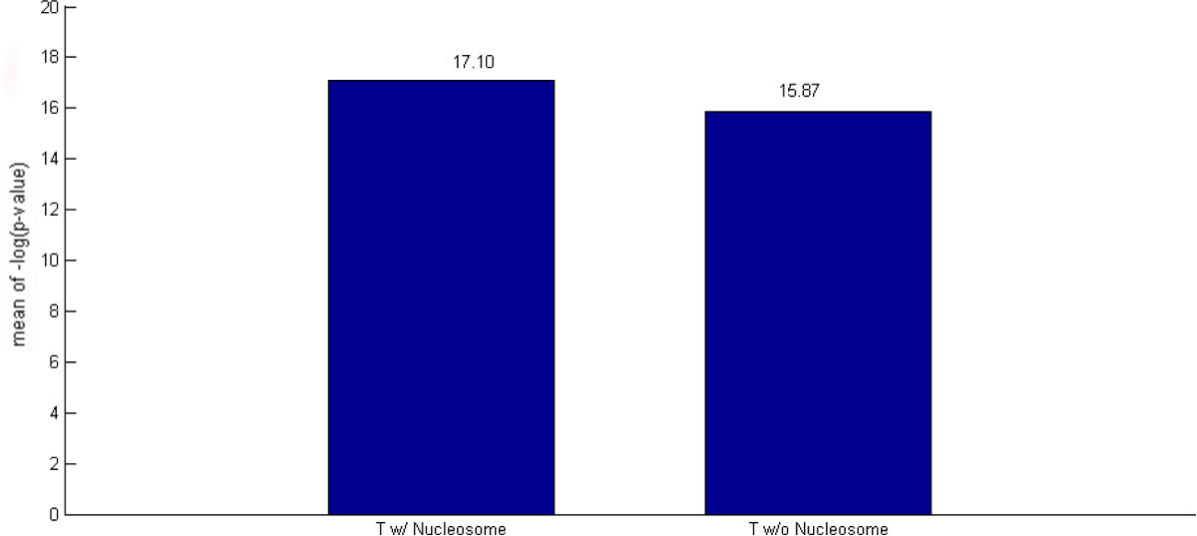
**The performance of our method with/without using nucleosome occupancy data**. To demonstrate that nucleosome occupancy data contributes to the overall improved prediction, we tested our method when nucleosome data are used (denoted as T w/ Nucleosome) and when nucleosome data are not used (denoted as T w/o Nucleosome) on the performance index 1. The figure shows that the *−logP *of T w/ Nucleosome is higher than that of T w/o Nucleosome, suggesting that nucleosome occupancy data do contribute to the overall improved prediction of our method.

To demonstrate that the nucleosome occupancy data contributes to the overall improved prediction, we tested our method when nucleosome data are used (denoted as T w/ Nucleosome) and when nucleosome data are not used (denoted as T w/o Nucleosome) on the performance index 1. As shown in Figure 6, the *−logP *of T w/ Nucleosome is higher than that of T w/o Nucleosome by 1.23, meaning that the p-value of T w/ Nucleosome is less than that of T w/o Nucleosome by more than 10 folds. This suggests that nucleosome occupancy data do contribute to the overall improved prediction of our method.

### Issue of applying our method to other model organisms

Our method is used to infer the cooperativity between two yeast TFs by integrating the TF-gene documented regulation, TFBS and nucleosome occupancy data. Theoretically, it can be applied to other model organisms which also have these three kinds of genome-wide data. There are two reasons why we used yeast as the model organism to test our method. First, yeast is the only organism which has more than 206000 TF-gene documented regulation data available. The useful TF-gene documented regulation data [29], which provide the experimentally validated regulatory relationships between TFs and genes, are collected from more than 1300 published papers by the team of the YEASTRACT database [18]. Second, yeast is the only organism that is tested by more than 10 existing algorithms. Therefore, using yeast as the model organism makes it possible to compare our predictions with the predictions of many existing methods.

### A cooperative TF Network

**Figure 7:**
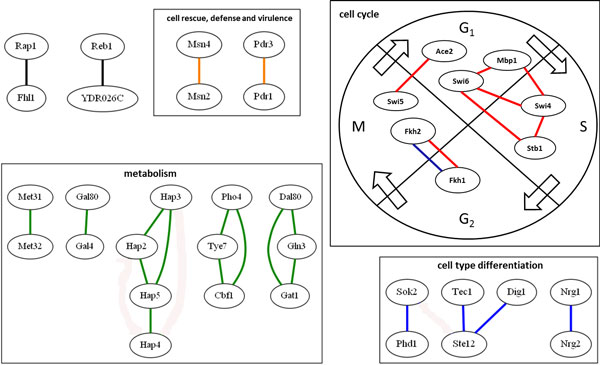
**A TF cooperativity network**. A TF cooperativity network is constructed from our 27 predicted cooperative TF pairs. Nodes represent TFs. A black line between two TFs means that these two TFs are predicted to be a cooperative TF pair but they are not annotated in the same MIPS functional category. A colored line between two TFs means that these two TFs are predicted to be a cooperative TF pair and they are annotated in the same MIPS functional category. The green color stands for metabolism, red for cell cycle, blue for cell type differentiation, orange for cell rescue, defense and virulence.

Figure 7 shows a cooperative TF network constructed from our 27 predicted cooperative TF pairs. This cooperative TF network has four main groups which belong to four biological processes according to MIPS functional categories. More precisely, there are (i) 12 pairs annotated in metabolism, (ii) 7 pairs annotated in cell cycle, (iii) 5 pairs annotated in cell type differentiation, and (iv) 2 pairs annotated in cell rescue, defense and virulence. This demonstrates that our method has the power to find cooperative TF pairs of different biological processes.

### Cell cycle

Since cell cycle process has been well investigated in the literature, let us discuss it in more details. Cell cycle is a complex process and it consists of four main phases: G1, S, G2 and M.

#### G1/S phase

The G1 to S phase transition of the eukaryotic cell cycle is crucial to the coordination of cell cycle progression with cellular growth. During the G1/S transition of the cell cycle in yeast, SBF and MBF are known to activate gene expression [30]. SBF is a protein complex composed of Swi4 and Swi6, and MBF is a protein complex composed of Mbp1 and Swi6 [31]. Our method successfully predicted the cooperativity between Swi4 and Swi6, and the cooperativity between Mbp1 and Swi6 (see Figure 7). Moreover, Mbp1 is known to related to Swi4 because the MBP1 SWI4 double knockout strain were inviable. The cooperativity between Mbp1 and Swi4 is successfully captured by our method (see Figure 7). In addition, a study suggests that Stb1 may affect MBF-dependent transcription [32]. Stb1 is a protein which regulates the timing of start transcription in the absence of the G1 regulator Cln3. The cooperativity between Stb1 and Swi6 (a member of MBF) is successfully identified by our method (see Figure 7).

#### G2/M phase

In the G2/M phase, Fkh1 and Fkh2 play essential roles in the activation of the CLB2 cluster genes and they share 72% identical DNA binding domain. Moreover, the double mutant of FKH1 and FKH2 displays obvious morphological change [33-36]. Our method successfully predicts the cooperativity between Fkh1 and Fkh2 (see Figure 7).

#### M/G1 phase

In M/G1 phase, Ace2 and Swi5 co-regulate the expression of many cell cycle genes in yeast [37]. Moreover, Ace2 and Swi5 proteins show similarity at the amino acid level and bind to the same DNA sequence with 82% identical DNA binding domains. Our method successfully identifies the cooperativity between Ace2 and Swi5 (see Figure 7).

## Conclusions

In this paper, we developed a method to infer the cooperativity between two TFs by integrating the TF-gene documented regulation, TFBS and nucleosome occupancy data. Two TFs are predicted as cooperative if (i) they have a significantly higher number of common target genes than random expectation and (ii) their binding sites (in the promoters of their common target genes) tend to be co-depleted of nucleosomes in order to make these binding sites simultaneously accessible to TF binding. A list of 27 cooperative TF pairs has been predicted by our method. Among these 27 predicted cooperative TF pairs, 19 pairs are also predicted by existing methods. The other 8 pairs are novel cooperative TF pairs. The biological relevance of these 8 novel cooperative TF pairs is justified by the existence of protein-protein interactions and co-annotation in the same MIPS functional categories. Moreover, our method is shown to outperform the 11 existing methods based on the three performance indices: (i) the similarity of protein-protein interaction partners between two TFs, (ii) the functional similarity between two TFs, and (iii) the overlap between a method's prediction result and the benchmark set of 27 known cooperative TF pairs. Finally, the cooperative TF network constructed from the 27 predicted pairs demonstrates that our method has the power to find cooperative TF pairs of different biological processes. We believe that our prediction will help biologists to understand the mechanism of transcriptional regulation in eukaryotic cells.

## Competing interests

The authors declare that they have no competing interests.

## Authors' contributions

WSW conceived the research topic and provided essential guidance. WSW, FJL and MHJ developed the algorithm and wrote the manuscript. FJL, MHJ and CCC performed all the simulations. YMH provided advices on the manuscript writing. All authors have read and approved the final manuscript.

## Declarations

The publication charges of this article were funded by Ministry of Science and Technology of Taiwan MOST-103-2221-E-006-174-MY2.
